# Human ClC-6 Is a Late Endosomal Glycoprotein that Associates with Detergent-Resistant Lipid Domains

**DOI:** 10.1371/journal.pone.0000474

**Published:** 2007-05-30

**Authors:** Sofie Ignoul, Jeannine Simaels, Diane Hermans, Wim Annaert, Jan Eggermont

**Affiliations:** 1 Laboratory of Membrane Transport, Department of Molecular Cell Biology, University of Leuven, Leuven, Belgium; 2 Laboratory for Membrane Trafficking, Department of Human Genetics, University of Leuven and V.I.B.11, Leuven, Belgium; University of Geveva, Switzerland

## Abstract

**Background:**

The mammalian CLC protein family comprises nine members (ClC-1 to -7 and ClC-Ka, -Kb) that function either as plasma membrane chloride channels or as intracellular chloride/proton antiporters, and that sustain a broad spectrum of cellular processes, such as membrane excitability, transepithelial transport, endocytosis and lysosomal degradation. In this study we focus on human ClC-6, which is structurally most related to the late endosomal/lysomal ClC-7.

**Principal Findings:**

Using a polyclonal affinity-purified antibody directed against a unique epitope in the ClC-6 COOH-terminal tail, we show that human ClC-6, when transfected in COS-1 cells, is N-glycosylated in a region that is evolutionary poorly conserved between mammalian CLC proteins and that is located between the predicted helices K and M. Three asparagine residues (N410, N422 and N432) have been defined by mutagenesis as acceptor sites for N-glycosylation, but only two of the three sites seem to be simultaneously N-glycosylated. In a differentiated human neuroblastoma cell line (SH-SY5Y), endogenous ClC-6 colocalizes with LAMP-1, a late endosomal/lysosomal marker, but not with early/recycling endosomal markers such as EEA-1 and transferrin receptor. In contrast, when transiently expressed in COS-1 or HeLa cells, human ClC-6 mainly overlaps with markers for early/recycling endosomes (transferrin receptor, EEA-1, Rab5, Rab4) and not with late endosomal/lysosomal markers (LAMP-1, Rab7). Analogously, overexpression of human ClC-6 in SH-SY5Y cells also leads to an early/recycling endosomal localization of the exogenously expressed ClC-6 protein. Finally, in transiently transfected COS-1 cells, ClC-6 copurifies with detergent-resistant membrane fractions, suggesting its partitioning in lipid rafts. Mutating a juxtamembrane string of basic amino acids (amino acids 71–75: KKGRR) disturbs the association with detergent-resistant membrane fractions and also affects the segregation of ClC-6 and ClC-7 when cotransfected in COS-1 cells.

**Conclusions:**

We conclude that human ClC-6 is an endosomal glycoprotein that partitions in detergent resistant lipid domains. The differential sorting of endogenous (late endosomal) versus overexpressed (early and recycling endosomal) ClC-6 is reminiscent of that of other late endosomal/lysosomal membrane proteins (e.g. LIMP II), and is consistent with a rate-limiting sorting step for ClC-6 between early endosomes and its final destination in late endosomes.

## Introduction

CLC proteins form an evolutionary conserved family of chloride channels and/or transporters that are expressed from bacteria to man [1]. The human genome contains 9 genes (CLCN1–7, CLCNKA, CLCNKB) that encode the pore-forming α-subunits (ClC-1 to -7, ClC-Ka and –Kb). In addition, auxiliary β-subunits that affect plasma membrane location or expression level of the α-subunit, have been described for ClC-Ka and –Kb (barttin) and ClC-7 (Ostm1) [2], [3]. More recently it has transpired that α-subunits can differ in terms of subcellular location (plasma membrane versus intracellular organelles) and mode of Cl^−^ transport (Cl^−^ channel versus Cl^−^/H^+^ antiporter) [4]–[7]. Consequently, the mammalian α-subunits can be classified in two subgroups, one functioning as plasma membrane Cl^−^ channels (ClC-1, -2, -Ka and –Kb) and another as intracellular Cl^−^/H^+^ antiporters (ClC-3 to -7). In mammals antiporter function has only been formally shown for ClC-4 and ClC-5 [5], [6], but the presence of a conserved glutamate corresponding to E203 in the E. coli ClC-ec1 that is responsible for H^+^-coupling of Cl^−^ transport [7], suggests a similar antiporter mode for ClC-3, ClC-6 and ClC-7. Some of the intracellular CLC's have been located in specific subcellular organelles: ClC-7 resides in late endosomes, lysosomes and the osteoclast resorption lacuna [8], ClC-5 in endosomes in the proximal tubule of the kidney [9], [10] and ClC-3 in (late) endosomes and synaptic vesicles [11]. Intracellular CLC's are thought to facilitate acidification of endosomal and lysosomal compartments by dissipating the lumen-positive membrane potential that arises from the electrogenic H^+^-transport by the V-type H^+^-ATPase [12]. Nevertheless, alternative functions have been proposed for intracellular CLC's, such as fusion of intracellular organelles [5] or trafficking of the endocytic receptor proteins megalin and cubulin [13].

In spite of being cloned more than 10 years ago [14] ClC-6 remains an enigmatic member of the mammalian CLC family. Sequence comparison shows ClC-6 to be most closely related to the late endosomal/lysosomal ClC-7 [14], but little is known about its function. Heterologous expression of ClC-6 either in *Xenopus* oocytes or in COS cells failed to generate specific membrane currents [14]–[16]. It should be added that in some instances membrane currents were recorded in ClC-6 expressing *Xenopus* oocytes, but identical currents were also observed in oocytes expressing the non-related pI_Cln_ protein and occasionally in control oocytes indicating that ClC-6 expression affected the expression of an endogenous anion channel [16], [17]. Very recently, it has been shown in a mouse model that loss of ClC-6 function leads to a lysosomal storage disease resembling neuronal ceroid lipofuscinosis [18].

In the present study we developed a specific antibody against human ClC-6, which recognizes the protein both in Western blotting and in immunofluorescence studies. This made it possible to determine the precise subcellular location of hClC-6 both endogenously in human neuronal SH-SY5Y cells and upon overexpression in COS-1 and Hela cells and to study its N-glycosylation pattern and its association with detergent resistant membranes.

## Materials and Methods

### Preparation of antiserum against human ClC-6

Rabbit antisera directed against human ClC-6 were raised against the synthetic peptide RKRSQSMKSYPSSEL (corresponding to residues 672–686 in hClC-6a) by Eurogentec (Seraing, Belgium). The peptide was COOH-terminally conjugated to hemocyamin and two rabbits were injected with the immunogen which consisted of an emulsion of the conjugate solution and Freund's adjuvant. Booster injections of the same immunogen with incomplete Freund's adjuvant were given 4 times at 4-week intervals. Both antisera were affinity-purified by the manufacturer.

### Preparation of the expression vectors

Human ClC-6a (hClC-6) cDNA was obtained as described [16]. The cDNA was cloned into the pCINeo/IRES-GFP [19] and the pcDNA3.1(−) (Invitrogen, Paisley, UK) expression vectors.

Mutants were made by overlap PCR [20] and involves the amplification of two overlapping mutant fragments (PCR 1 and 2), followed by amplification of the overlap fragment (PCR 3). Reaction conditions were as follows for PCR 1 and 2: initial denaturation at 94°C for 5 min, 30 cycles of denaturation at 94°C for 30 s, annealing at 60°C for 1 min, extension at 72°C for 10 min with a final extension at 72°C for 20 min. For PCR 3 the PCR parameters were similar to those of PCR 1 and 2, except the annealing temperature was augmented gradually between 50°C and 68°C during the 30 cycles. The PCR products were visualized by ethidium bromide staining of a 1% agarose gel. The overlap fragment was eluted from the gel with the GenElute™ Gel Extraction Kit (Sigma-Aldrich, St. Louis, MO, USA) and ligated into the pcDNA3.1(−) expression vector using BamHI and HindIII restriction sites. Mutations were verified using dye terminator-based sequencing (DYEnamic ET Terminator Cycle Sequencing Kit, Amersham Biosciences, Piscataway, NJ, USA) on an automated MegaBACE sequencer (Amersham Biosciences, Piscataway, NJ, USA).

Human ClC-7 was expressed with a pQBI/GFP-hClC-7 vector.

Expression vectors of COOH-terminally GFP fusions of Rab4, Rab5, Rab7 and Rab11 proteins are pEGFP-C3 vectors as described [21].

### Cell culture and transfection

The human neuroblastoma SH-SY5Y cell line was obtained from American Tissue Type Culture Collection CRL 1650 (Besthesda, MD, USA). Cells were grown in Dulbecco's modified Eagle's medium supplemented with 15% (v/v) fetal calf serum (FCS), 1% glutamax; 1% (v/v) non-essential amino acids, 100 units/ml penicillin and 100 µg/ml streptomycin. Cells were incubated in a humified incubator at 5% CO_2_ and 37°C. From day 1 after seeding, cells were differentiated in the presence of 10 µM all-*trans*-retinoic acid (RA, Sigma-Aldrich) in cell medium containing 1% FCS in the absence of light. After 6 days, the medium was replaced by cell medium without FCS, containing 2 nM brain-derived neurotrophic factor (BDNF, Sigma-Aldrich). After 48 hours differentiated cells were used for further experiments.

Transfections of differentiated SH-SY5Y were performed after 6 days of differentiation with retinoic acid (RA) using Lipofectamine™ 2000 transfection reagent (Invitrogen) as described in the manufacturer's protocol. After 24 hours, the medium was replaced by cell medium without FCS, containing 2 nM BDNF and cells were used for further experiments after 48 hours.

COS-1 SV 40 African monkey kidney cells, and HeLa epithelial cells from an epidermoid carcinoma of the human cervix, were cultured in Dulbecco's modified Eagle's medium supplemented with 10% (v/v) fetal calf serum, 3.8 mM L-glutamine, 0.9% (v/v) non-essential amino acids, 85 units/ml penicillin and 85 µg/ml streptomycin. COS-1 cells were incubated in a humified incubator at 9% CO_2_ and 37°C. HeLa cells were incubated in a humified incubator at 5% CO_2_ and 37°C.

COS-1 and HeLa cells were transiently transfected with expression vectors using Gene-Juice® transfection reagent (Novagen, Darmstadt, Germany) as described in the manufacturer's protocol. Transfections were performed the day after seeding.

### Membrane preparation

Microsomes from transfected COS-1 cells were prepared as described by Verbomen *et al.* [22].

Protein concentrations were determined by the bicinchonic acid method (Pierce, Rockford, IL, USA).

### Immunocytochemistry

SH-SY5Y cells, COS-1 and HeLa cells were grown on gelatine coated coverslips. SH-SY5Y cells were differentiated as described (see supra) and COS-1 and HeLa cells were transiently transfected with different constructs. Cells were fixed in 3.7% paraformaldehyde for 15 min at room temperature and permeabilized with 0.2% Triton X-100 for 2 min at room temperature. Non-specific binding was blocked by incubation for 5 h in PBS containing 3% BSA. Primary antibodies were diluted in 3% BSA in PBS and incubated overnight at 4°C. Immunofluorescence was performed using the following primary antibodies: rabbit anti-hClC-6 (1∶1000; Eurogentec), mouse anti-EEA-1 (Clone 14, 1∶150; BD Biosciences, Erembodegem, Belgium), mouse anti-transferrin receptor (Clone H68.4, 1∶100; Invitrogen), mouse anti-LAMP-1 (H5G11, 1∶100; Santa Cruz, California, USA), mouse anti-human Golgin-97 (CDF4, 1:200, Molecular Probes, Leiden, The Netherlands), mouse anti-KDEL (recognizes BIP, Clone 10C3, 1∶100; Stressgen Bioreagents, AM Uden, The Netherlands). EEA-1, transferrin receptor, LAMP-1, Golgin-97, and BIP proteins function as markers for early endosomes, recycling endosomes, late endosomes/lysosomes, Golgi and ER, respectively. Secondary antibodies were added in 3% BSA in PBS and incubated for 1 h at room temperature. Secondary antibodies were goat anti-rabbit Alexa Fluor 488 or 594, or goat anti-mouse Alexa Fluor 594 (Molecular Probes). Finally, the coverslips were mounted in Vectashield (Vector Laboratories, Brussels, Belgium) to inhibit photobleaching and nuclei were visualized by adding TO-PRO®-3 iodide (1∶1000; Molecular Probes) to the mounting medium. Samples were viewed by confocal laser scanning microscopy (CLSM) using a Zeiss Radiance 2100 (Zeiss, Jena, Germany) coupled to an upright Nikon Eclipse E800 upright microscope (objective 60×, planAPO). Immunofluorescence data were acquired using Lasersharp2000 (Zeiss) and finally processed by Adobe Photoshop.

### Preparation of detergent resistant membrane fractions (DRM)

DRM fractions were prepared from transfected COS-1 cells as described [23]. Cells were washed twice with PBS and lysed for 1 h on ice in excess (10-fold excess (w/w) over protein), ice-cold Triton X-100 (1%) buffer containing 25 mM Tris (pH 7.4), 100 mM NaCl, 90 mM Mannitol, 1 mM EGTA, 2 mM DDT and protease inhibitor cocktail (Sigma Aldrich). The lysate was separated by upward flotation on a sucrose gradient as described earlier. Upward flotation of DRM's was verified by Western blotting and immunostaining with a monoclonal anti-caveolin-1 antibody (1/1000; BD Biosciences) and a polyclonal anti-Fyn antibody (FYN3, 500 ng/ml; Santa Cruz; data not shown). All blots were tested by immunostaining with a monoclonal anti-transferrin receptor antibody (1 µg/ml; Invitrogen) as a negative control. Fractions were tested for distribution of hClC-6 (wild type and AAGAA-mutant) by Western blotting and immunostaining with the polyclonal α-hClC-6 antibody (1∶1000). DRM fractions were also prepared from GFP-hClC-7 overexpressing COS-1 cells and immunostained for caveolin-1, transferrin receptor and GFP (GFP Monoclonal Antibody, 1∶500; Clontech).

### Deglycosylation studies

Digestions with Peptide N-glycosidase F (PNGaseF, New England Biolabs, Ipswick, MA, USA) which removes both core (mannose-rich) and complex (trimmed and modified) glycans or with Endoglycosidase H (EndoH, New England Biolabs) which removes core but not complex glycans, were performed as advised by the supplier on 20 µg of glycoprotein with a preliminary denaturation step during 24 hours on 37°C. Tunicamycin (Sigma-Aldrich) which blocks the first step in the N-glycosylation process (transfer of the mannose-rich core glycan from the dolichol carrier to an asparagine acceptor), was added 4 hours after transient transfection of the COS-1 cells with hClC-6a WT or mutants in a final concentration of either 0.05 or 0.1 µg/ml during a 36-h period.

### SDS PAGE and Western-Blot analysis

Microsomes from COS-1 cells, transiently transfected with the different constructs, were analysed by NuPAGE™ 4–12% (v/v) Bis-Tris SDS-PAGE gels using MOPS-buffer (Invitrogen), following the manufacturer's protocol. After electrophoresis, the separated proteins were transferred onto a PVDF membrane (Immobilon-P; Millipore, Bedford, MA, USA) by semi-dry electroblotting. The blots were blocked overnight at 4°C in PBS containing 0.1% (v/v) Tween-20 and 5% (w/v) non-fat dry milk powder. The blots were incubated with the primary antibody and subsequently with the horseradish peroxidase (HRP) conjugated secondary antibody. The immunoreactive bands were visualized with SuperSignal® West Pico Chemiluminescent Substrate (Pierce) and exposed to HyperFilm. The HyperFilm was developed using a KODAK X-Omat 1000 (KODAK, Rochester, NY, USA).

## Results

### The polyclonal α-hClC-6 antibody recognizes human ClC-6 (hClC-6) in transiently transfected cells

**Figure 1:**
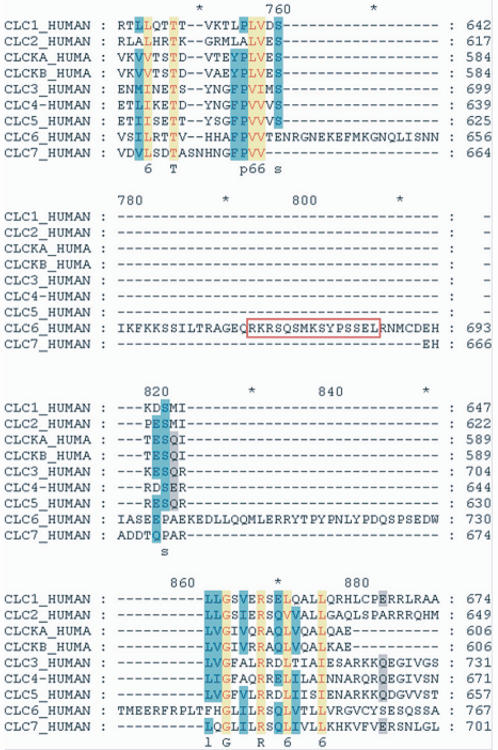
Development of a polyclonal antibody against human ClC-6 (hClC-6). Multiple sequence alignment (ClustalW) of all human CLC proteins revealed a COOH-terminal region that is unique for ClC-6 (aa 639–740). This region interrupts the first cystathionine-β synthase (CBS) domain in the COOH-terminal cytosolic tail [52]. Polyclonal antibodies were raised against an epitope (aa 672–686; red box) in this unique region.

**Figure 2:**
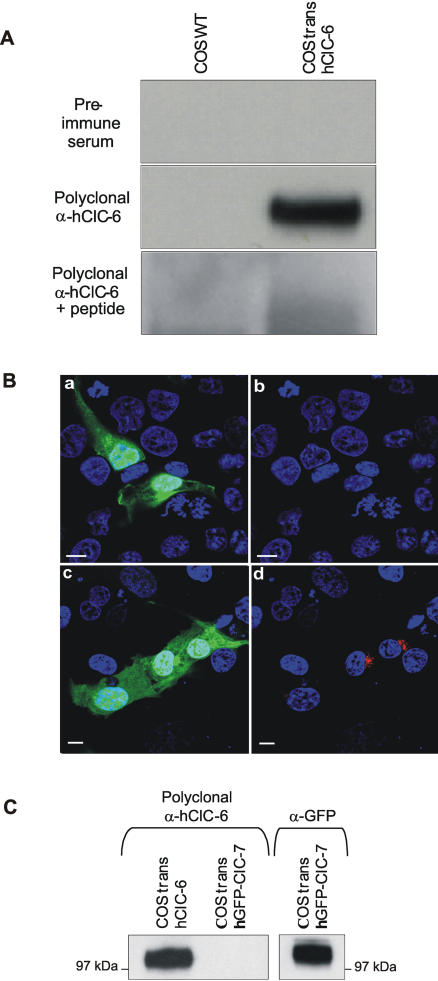
Characterization of the polyclonal α-hClC-6 antibody. (A) Western blotting of microsomal membranes of wild-type COS-1 cells and COS-1 cells transiently transfected with pcDNA3.1(−)/hClC-6a. Incubation with the affinity-purified polyclonal α-hClC-6 resulted in a band of approximately 100 kDa in transfected COS-1 cells, but not in wild type COS-1 cells. Such a band was not observed in transfected COS-1 cells when the blot was incubated with pre-immune serum or when the polyclonal α-hClC-6 was preincubated with the epitope-peptide before applying to the blot. (B) Immunofluorescence of COS-1 cells transfected with the bicistronic vector pcINeo/GFP-IRES/hClC-6a which ensures strict coupling between GFP expression (green signal in panels a and c) and ClC-6 expression. Incubation with pre-immune serum (panel b) never generated a positive signal in transfected cells. In contrast, a positive signal for ClC-6 (red in panel d) was exclusively observed in transfected cells incubated with polyclonal α-hClC-6. Nuclei were counterstained with blue TO-PRO®-3. The scale bar corresponds to 10 µm. (C) Western blot of microsomal membrane fractions from transiently transfected COS-1 cells expressing either hClC-6 or GFP-hClC-7. Incubation with α-hClC-6 generated a positive signal in the hClC-6 expressing membrane fractions, but not in the hClC-7 expressing membranes. Expression of GFP-hClC-7 was confirmed by incubation with a monoclonal α-GFP antibody.

A short peptide (amino acids 672–686) in the COOH-terminal cytosolic tail of hClC-6 was selected to raise affinity-purified polyclonal antibodies against hClC-6 (Fig. 1). The antibodies were first tested on Western blots using microsomal membrane fractions of COS-1 cells, either wild type or transiently transfected with a hClC-6 expression vector (Fig. 2A). Incubation with the polyclonal α-hClC-6 antibody resulted in a strong band of approximately 100 kDa (theoretical molecular mass of unglycosylated hClC-6 is 96 kDa, but see further) in hClC-6 expressing COS-1 cells, but not in untransfected wild type COS-1 cells (Fig. 2A). A similar result was obtained in transfected HeLa cells (data not shown). The specificity of the α-hClC-6 antibody was confirmed by incubating Western blots with pre-immune serum or α-hClC-6 antibody pre-adsorbed to the epitope peptide. The pre-immune serum failed to visualize the 100 kDa band in hClC-6 expressing COS-1 cells, whereas pre-adsorption caused a nearly complete disappearance of the 100 kDa band (Fig. 2A). Subsequently the α-hClC-6 antibody was tested for immunofluorescence experiments (Fig. 2B). Therefore, COS-1 cells transiently transfected with a bicistronic GFP/hClC-6 expression vector [19] were incubated with pre-immune serum (Fig. 2Ba–b) or the α-hClC-6 antibody (Fig. 2Bc–d). Specific staining was only observed with the α-hClC-6 antibody and was exclusively associated with GFP-expressing cells.

Next we tested whether the α-hClC-6 antibody cross-reacted with hClC-7 as ClC-6 is most closely related to ClC-7 [14]. To do so, COS-1 cells were transiently transfected with a GFP-hClC-7 expression vector that encodes human ClC-7 with a GFP fused at the NH_2_-terminus. Although ClC-7 expression levels were high, as shown by the Western blot using anti-GFP antibody, no crossreactivity was found for the α-hClC-6 antibody (Fig. 2C). We therefore conclude that the polyclonal α-hClC-6 antibody specifically recognizes hClC-6 when transiently expressed in COS-1 cells, both on Western blot and indirect immunofluorescence experiments.

### hClC-6 is N-glycosylated on multiple positions

**Figure 3:**
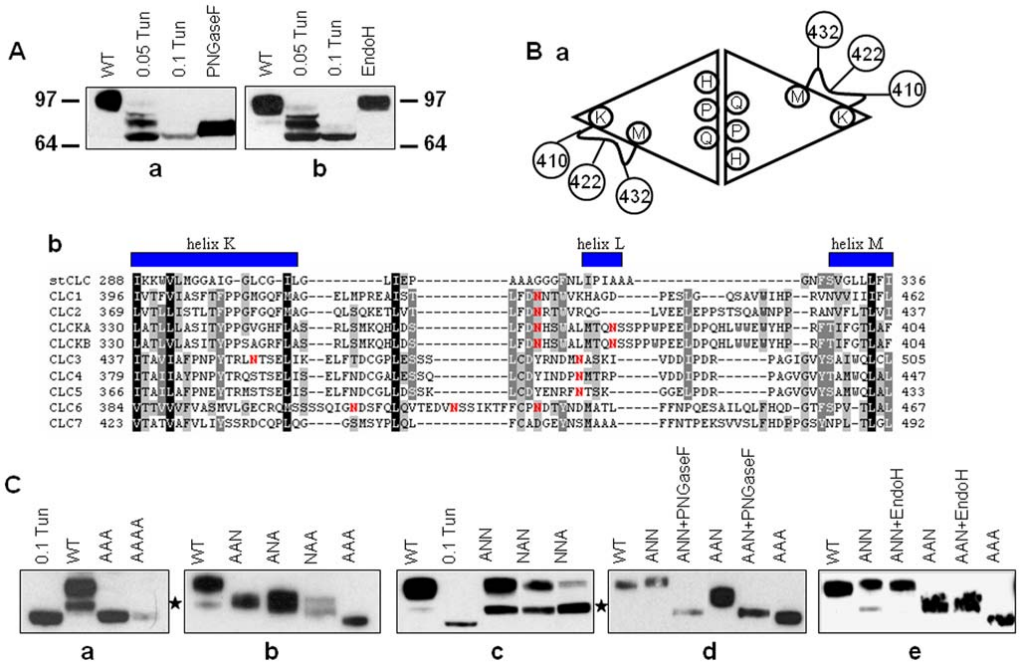
hClC-6 is glycosylated upon overexpression. (A) Western blots showing the effect of tunicamycin and (a) PNGaseF and (b) EndoH on hClC-6. For tunicamycin COS-1 cells were incubated with tunicamycin (0.05 and 0.1 µg/ml) for 36 hours; for PNGaseF and EndoH membrane fractions of hClC-6 expressing COS-1 cells were treated as described in materials and methods. ‘WT’ refers to untreated hClC-6. The small difference in molecular mass between PNGaseF and tunicamycin-treated hClC-6 might be due to the presence of oligosaccharides carrying fucose-linked α1–3 to the GlcNac attached directly to asparagines, which are PNGaseF resistant as described by Dwek *et al.* [53]. (B)(a), Model of the hClC multimeric structure of 2 homologous subunits with the possible glycosylation sites marked as spheres. (b), Partial sequence alignment (ClustalW) of all members of the CLC family reveals that Asn residues that possibly participate in glycosylation (marked in red) in hClC-6 are situated in a region that is poorly conserved among the other members of the CLC family and located between predicted helices K and M. (C) Western blots of membrane fractions of COS-1 cells expressing WT or mutant hClC-6. (a), Glycosylation pattern of the triple (AAA: N410A/N422A/N432A) and quadruple (AAAA: N137A/N410A/N422A/N432A) mutant compared to WT and WT with tunicamycin. (b), Glycosylation pattern of the double mutants (AAN: N410A/N422A; ANA: N10A/N432A; NAA: N422A/N432A) compared to glycosylated WT and the triple mutant (AAA). (c), Glycosylation pattern of the single mutants (ANN: N410A, NAN: N422A, NNA: N432A) compared to the glycosylated WT and deglycosylated WT treated with tunicamycin. (d), Effect of PNGaseF treatment on a single (ANN: N410A) and double (AAN: N410A/N422A) mutant compared to WT and triple (AAA) mutant. (e), Effect of EndoH treatment on a single (ANN: N410A) and double (AAN: N410A/N422A) mutant compared to WT and triple (AAA) mutant. Bands marked with an asterisk, occasionally observed in the WT protein and frequently observed in single mutants after sustained exposure represent possible intermediary biosynthetic products, which are PNGaseF-sensitive and EndoH-insensitive as shown in panels (d) and (e).

Incubation of transfected COS-1 cells with tunicamycin (0.1 µg/ml) significantly increased the mobility of hClC-6 (65 kDa as compared to 100 kDa) demonstrating that it is N-glycosylated (Fig. 3A). At a lower concentration (0.05 µg/ml) tunicamycin induced the appearance of several intermediate bands between 100 and 65 kDa indicating multiple glycosylation of hClC-6 (see below). A similar reduction in molecular mass was observed when hClC-6 was treated with PNGaseF (Fig. 3Aa). In contrast, EndoH did not affect the electrophoretic mobility of hClC-6 (Fig. 3Ab). The tunicamycin- and PNGaseF-sensitivity in combination with the EndoH resistance indicates that hClC-6 carries complex N-glycans that have been processed and modified in the Golgi apparatus. Furthermore, there is a discrepancy between the apparent molecular mass on SDS-PAGE (100 kDa for glycosylated hClC-6 and 65 kDa for non-glycosylated hClC-6) and the predicted molecular mass (97 kDa for the non-glycosylated protein). Proteolytic cleavage of hClC-6 was excluded since the same band was detected by the α-hClC-6 antibody which recognizes an epitope in the COOH-terminal tail, and an anti-Myc antibody directed at a Myc epitope tag inserted at the hClC-6 NH_2_-terminus (data not shown). The faster migration pattern on SDS-PAGE most likely reflects anomalous migration as has also been reported for ClC-5 [24].

We then proceeded to identify the N-glycosylated asparagine residues in hClC-6. Sequence analysis of hClC-6 revealed 7 potential N-glycosylation motifs (N-X-[S,T] with X any amino acid except proline). Modeling of hClC-6 on the crystal structure of prokaryote ClC indicated that four asparagines, i.e. N137, N410, N422 and N432, were located in an exoplasmic loop or at the exoplasmic end of a membrane helix and are therefore positioned at the correct topological position for N-glycosylation: N137 is located at the exoplasmic end of helix C and N410, N422, N432 are located in an exoplasmic region between helices K and M (Fig. 3B). To find out which asparagines are N-glycosylated these residues were mutated to alanine, either individually or in group. The quadruple mutant (AAAA-hClC-6: N137A/N410A/N422A/N432A) and the triple mutant (AAA-hClC-6: N410A/N422A/N432A) migrated on SDS-PAGE with the same mobility as non-glycosylated hClC-6 (tunicamycin treatment; Fig. 3Ca). Since there was no difference between AAA-hClC-6 and AAAA-hClC-6, it appears that N137 is not glycosylated and that N-glycosylation is limited to the asparagine residues in the region between helices K and M. This was tested by introducing single and double mutations for N410, N422 and N432. All double mutants (NAA-hClC-6: N422A/N432A; ANA-hClC-6: N410A/N432A; AAN-hClC-6: N410A/N422A) were glycosylated as indicated by their higher apparent molecular mass than non-glycosylated hClC-6 and by their PNGaseF sensitivity (Fig. 3Cb and 3Cd). Importantly, wild type hClC-6 migrated slower than the double mutants which is consistent with hClC-6 carrying more than one glycan moiety. This was confirmed by the analysis of the single mutants (NNA-hClC-6: N432A; NAN-hClC-6: N422A; ANN-hClC-6: N410A, Fig. 3Cc and 3Cd) which contain two potential N-glycosylation sites. These were all PNGaseF-sensitive and migrated slower than the (monoglycosylated) double mutants which is compatible with the addition of a second glycan. However, wild type hClC-6 and the single mutants migrated at the same position indicating that in wild type hClC-6 only 2 of the 3 potential glycosylation sites are effectively used.

### Endogenous hClC-6 colocalizes with LAMP-1 in a human neuronal SH-SY5Y cell line

**Figure 4:**
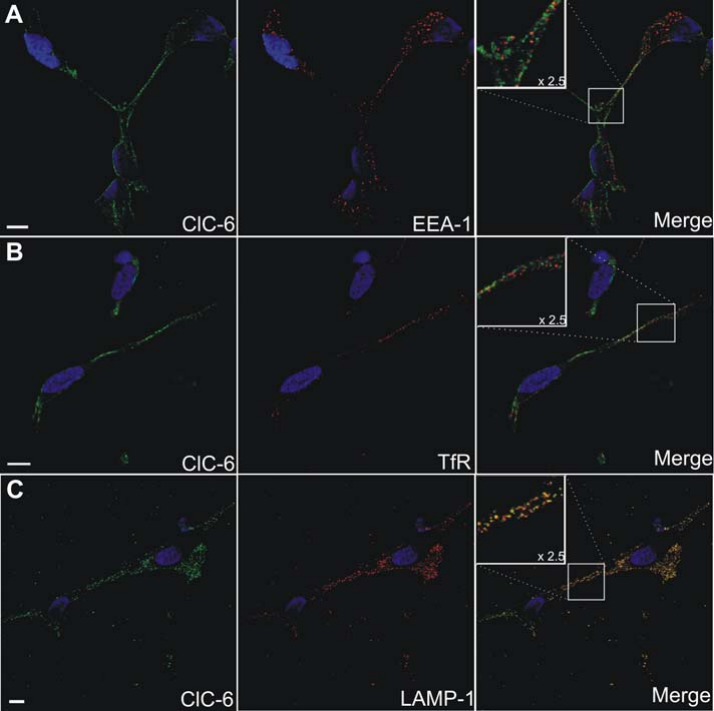
Immunolocalization of endogenous hClC-6 in SH-SY5Y cells. Double immunofluorescence confocal images of SH-SY5Y cells. hClC-6 (left column) was detected using the polyclonal α-hClC-6 antibody and visualized with anti-rabbit IgG antibodies conjugated to Alexa Fluor 488 (green signal). Markers for different endosomal compartments (middle column) were (A) mouse anti-EEA-1 (an early endosome marker); (B) mouse anti-transferrin receptor (TfR, an early/recycling endosome marker); (C) mouse anti-LAMP-1 (a late endosomal/lysosomal marker). The marker antibodies were visualized by anti-mouse IgG antibodies conjugated to Alexa Fluor 594 (red signal). In the merged pictures (right column) colocalization is indicated by a yellow signal. The scale bars represent 10 µm.

A crucial question with respect to the intracellular CLC's deals with their specific subcellular location. We therefore examined by means of confocal laser scanning microscopy (CLSM) the subcellular distribution of endogenous human ClC-6 in a differentiated neuronal cell line SH-SY5Y cells (Fig. 4). ClC-6 displays a punctuated pattern that is present both around the nucleus in the cell body and in the neuronal outgrowths. There is no substantial overlap with the early endosomal marker EEA-1 (Fig. 4A) nor with transferrin receptor (TfR; Fig. 4B), a marker for recycling endosomes [25]. However, endogenous ClC-6 strongly colocalized with LAMP-1 (a marker for late endosomes/lysosomes) both perinuclearly and in the cell periphery (Fig. 4C). This is in agreement with Poët *et al.* [18] who have recently reported that in mouse brain sections ClC-6 mainly colocalizes with LAMP-1 and concluded that ClC-6 resides in a late endosomal compartment.

### Immunolocalization of hClC-6 in transiently transfected COS-1 and HeLa cells

**Figure 5:**
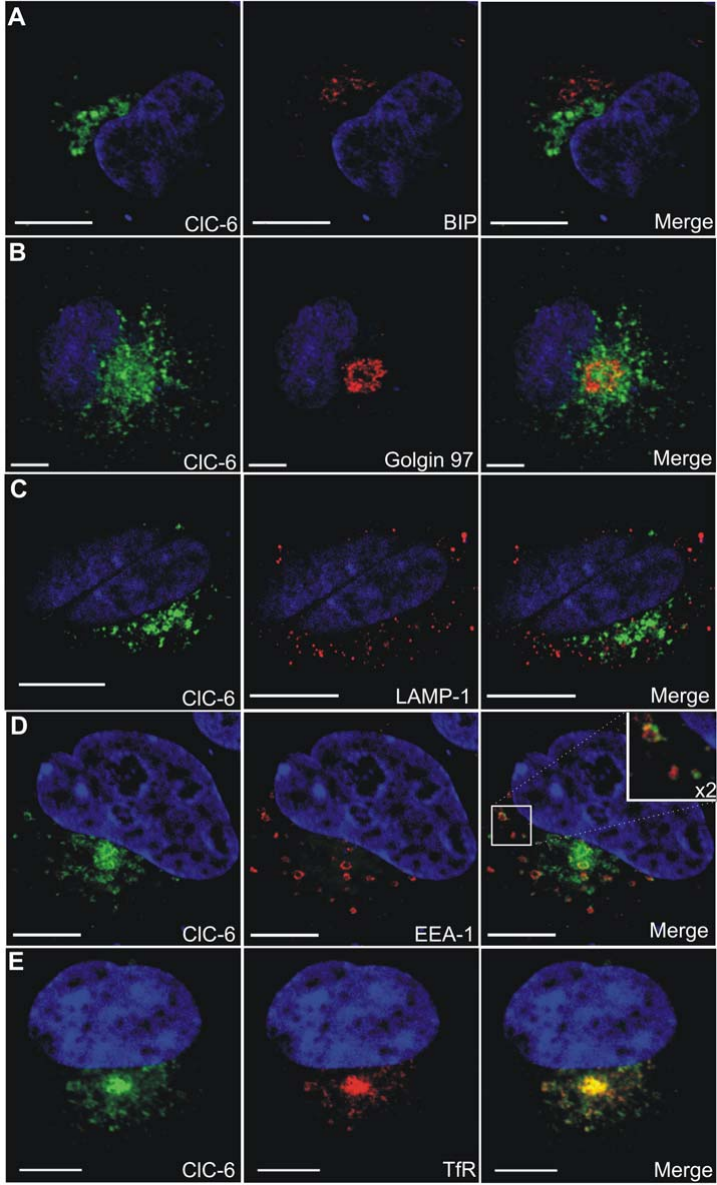
Immunolocalization of hClC-6 in transfected COS-1 cells. Double immunofluorescence confocal images of COS-1 cells transiently transfected with pcDNA3.1(−)/hClC-6a. hClC-6 expression (left column) was detected using the polyclonal α-hClC-6 antibody and visualized with anti-rabbit IgG antibodies conjugated to Alexa Fluor 488 (green signal). Organelles were stained with the following antibodies or markers (middle column): (A) mouse anti-BIP (an endoplasmic reticulum marker); (B) mouse anti-Golgin-97 (a Golgi marker); (C) mouse anti-LAMP-1 (a late endosomal/lysosomal marker); (D) mouse anti-EEA-1 (an early endosome marker); (E) mouse anti-transferrin receptor (TfR, an early/recycling endosome marker). The marker antibodies were visualized by anti-mouse IgG antibodies conjugated to Alexa Fluor 594 (red signal). The column on the right shows merged pictures of ClC-6 expression and marker staining with yellow indicating colocalization. The scale bars represent 10 µm.

Complementary experiments with respect to the subcellular localization of hClC-6 were performed in transiently transfected COS-1 cells (Fig. 5). Typically hClC-6 displayed a perinuclear staining pattern often residing in relatively large (a few micrometer in diameter) vesicular structures (Fig. 5). The distribution pattern of hClC-6 clearly did not overlap with endoplasmic reticulum (BIP, Fig. 5A) nor with the Golgi markers Golgin-97 (Fig. 5B) or GM130 (not shown). A partial overlap with Golgin-97 was observed in a few transfected cells. In contrast to endogenous ClC-6 in SH-SY5Y cells, transiently expressed hClC-6 did not overlap with LAMP-1 in COS-1 cells (Fig. 5C), but it showed substantial colocalization with markers for early endosomes (EEA-1) or the recycling pathway (TfR).

**Figure 6:**
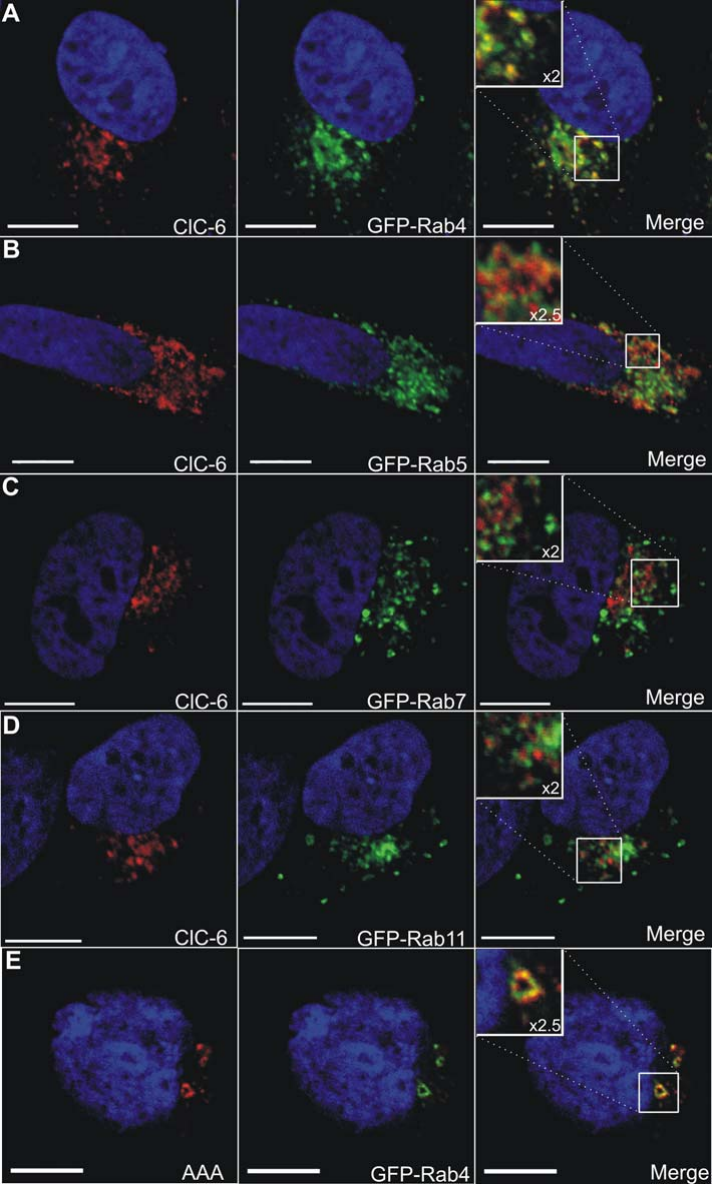
Colocalization of overexpressed hClC-6 with different endosomal Rab-proteins. Confocal images of double transiently transfected HeLa cells, expressing hClC-6 and (A) GFP-Rab4, (B) GFP-Rab5, (C) GFP-Rab7, (D) GFP-Rab11. hClC-6 was detected with the polyclonal α-hClC-6 antibody and visualized with anti-rabbit IgG antibodies conjugated to Alexa Fluor 594. The Rab signals were visualized by the GFP signal. (E) Represents a confocal image of double transiently transfected COS-1 cells, expressing the glycosylation-deficient AAA-hClC-6 (red signal) and GFP-Rab4 (green signal). The scale bars represent 10 µm.

The endosomal localization was further dissected via co-expression of hClC-6 with Rab4, Rab5, Rab7 and Rab11 which are established marker proteins for early endosomes (Rab5) [26], [27], late endosomes/lysosomes (Rab7) [28] and recycling endosomes (Rab4 and Rab11) [29]–[32]. Because of the better morphology, these experiments were conducted in HeLa cells co-expressing hClC-6 and a GFP-Rab fusion protein using CLSM (Fig. 6), but similar data were acquired in COS-1 cells (data not shown). From panels C and D in Fig. 6 it is clear that little or no overlap was found with Rab7 (Fig. 6C) nor with Rab11 (Fig. 6D). For Rab7 this was not surprising given the lack of colocalization with LAMP-1 (see above). However, there was partial colocalization with Rab5 (Fig. 6B) and an even better overlap with Rab4 (Fig. 6A). Thus, during transient overexpression in COS or HeLa cells hClC-6 ends up in an endosomal compartment that is positive for early endosomal markers (EEA-1 and Rab5) and a subset of recycling endosomal markers (TfR and Rab4; see Discussion for a further description of this compartment). In this respect it should be pointed out that not only Rab11-positive, but also Rab4-positive endosomes are found in the perinuclear region [21] which would account for the perinuclear signal of overexpressed hClC-6.

We also investigated whether N-glycosylation is required for endosomal location of hClC-6 in transfected HeLa cells. CLSM of the glycosylation-deficient AAAA-hClC-6 (data not shown) and AAA-hClC-6 showed substantial colocalization with Rab4 (Fig. 6E) indicating that glycosylation is not essentially required for delivery to the endosomal compartment. A similar overlapping pattern with Rab4 was observed for the single and double N-glycosylation mutants (data not shown).

### Immunolocalization of overexpressed hClC-6 in the neuronal SH-SY5Y cell line

Since the expression pattern of overexpressed hClC-6 in COS-1 and HeLa cells differed from the endogenous hClC-6 distribution in SH-SY5Y neuronal cells, we investigated whether this discrepancy reflects cell type-specific differences in protein sorting in the endosomal system or, alternatively, whether this is the result of overexpression.

**Figure 7:**
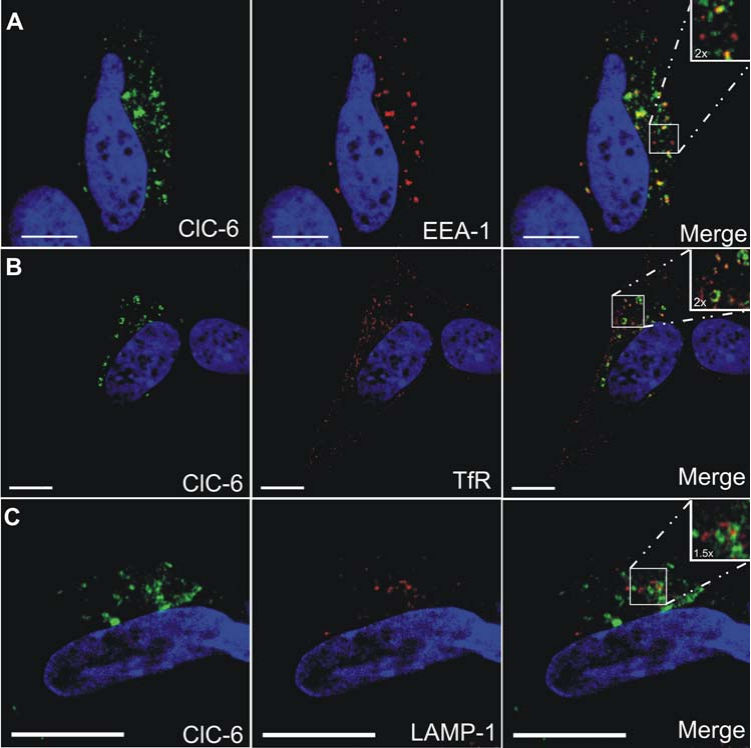
Immunolocalization of overexpressed hClC-6 in SH-SY5Y cells. Double immunofluorescence confocal images of SH-SY5Y cells, transiently transfected with pcDNA3.1(−)/hClC-6a expression vector. Overexpression levels were very high, so that transfected cells could easily be distinguished from non-transfected cells. Overexpressed hClC-6 (left column) was detected using the polyclonal α-hClC-6 antibody and visualized with anti-rabbit IgG antibodies conjugated to Alexa Fluor 488 (green signal). Markers for different endosomal compartments (middle column) were (A) mouse anti-EEA-1 (an early endosome marker); (B) mouse anti-transferrin receptor (TfR, an early/recycling endosome marker); (C) mouse anti-LAMP-1 (a late endosomal/lysosomal marker). Primary antibodies were visualized using anti-mouse IgG antibodies conjugated to Alexa Fluor 594 (red signal). In the merged pictures (right column) colocalization is indicated by a yellow signal. The scale bars represent 10 µm.

Therefore, we transiently transfected differentiated SH-SY5Y cells with an hClC-6 expression vector and determined the distribution of overexpressed hClC-6 by means of CLSM. The overexpression levels in transfected cells were very high, so that transfected cells could easily be distinguished from non-transfected cells. As in COS-1 and HeLa cells, the overexpressed hClC-6 displayed a perinuclear staining pattern (Fig. 7). This pattern partially overlapped with the early endosomal marker EEA-1 (Fig. 7A) and recycling endosomal pathway marker TfR (Fig. 7B), but not with the late endosomal marker LAMP-1 (Fig. 7C). Furthermore, after cotransfection of hClC-6 with a Rab4-GFP expression vector we observed a high degree of colocalization, analogous to overexpression in COS-1 and HeLa cells (data not shown). Thus, although SH-SY5Y cells can sort endogenous ClC-6 to a LAMP-1 positive compartment, hClC-6 does not reach this compartment upon overexpression.

### hClC-6 resides in detergent resistant membrane fractions in transiently transfected COS-1 cells

**Figure 8:**
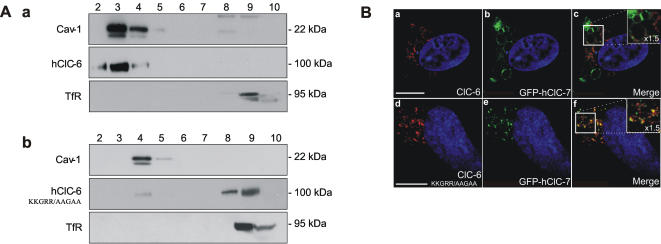
hClC-6 resides in detergent resistant membrane fractions. (A) DRM fractions of COS-1 cells overexpressing respectively (a) hClC-6 and (b) KKGRR/AAGAA-hClC-6; were prepared and separated on a sucrose gradient. Upward flotation of the DRM's was checked by distribution of caveolin-1 (Cav-1) which migrated to the top of the gradient (fractions 2/3/4). Transferrin receptor (TfR), a non-raft membrane protein, was used as a negative control (fractions 8/9 at the bottom of the gradient). hClC-6 expression was checked by staining with the polyclonal α-hClC-6. (B) Confocal images of double transiently transfected COS-1 cells, expressing GFP-hClC-7 and wild type hClC-6 (panels a to c) or KKGRR/AAGAA-hClC-6 (panels d to f). Wild type and KKGRR/AAGAA-hClC-6 were detected with the polyclonal α-hClC-6 antibody and visualized with anti-rabbit IgG antibodies conjugated to Alexa Fluor 594 (red signal, panels a and d). ClC-7 expression is visualized by the GFP signal (green signal, panels b and e). A yellow signal indicates colocalization (panels c and f). Scale bars represent 10 µm.

In a final series of experiments we investigated whether hClC-6 associates with detergent resistant membrane (DRM) fractions. Transiently transfected COS-1 cells were lysed with Triton X-100 at 4°C and DRM's were separated by flotation on a sucrose gradient. Western blot analysis of the gradient fractions (equal amount of volume) showed that overexpressed hClC-6 co-distributed with caveolin-1 in the upper part of the sucrose gradient corresponding to the DRM fractions (Fig. 8A). In contrast, the transferrin receptor which does not associate with detergent resistant membranes [33], did not float upwards in the sucrose gradient (Fig. 8A). It has been shown that DRM association of CD4, an intrinsic membrane protein, critically depends on a cytosolic, membrane-proximal stretch of positively charged amino acids (RHRRR) [34]. Intriguingly, hClC-6 contains a similar positively charged sequence KKGRR (amino acids 71–75) immediately upstream and at the cytosolic side of the first transmembrane helix B. Indeed, mutation of KKGRR into AAGAA disrupted the DRM association (Fig. 8Ab). The large majority of AAGAA-hClC-6 failed to float upwards and remained at the bottom of the sucrose gradient together with the transferrin receptor.

Strikingly, the KKGRR/AAGAA mutation also affected the segregation of ClC-6 and ClC-7. Although Suzuki *et al.* reported a significant colocalization between ClC-6 and ClC-7 cotransfected in HEK293 cells [35], we observed a clear separation of both CLC proteins when cotransfected in COS-1 cells. Indeed, wild-type hClC-6 and ClC-7 resided either in different vesicles or in different microdomains of the same vesicle upon cotransfection (Fig 8Ba–c). However, the distribution of AAGAA-hClC-6 and ClC-7 overlapped substantially as shown by the yellow pattern in the merged panel (Fig. 8Bd–f) suggesting (partial) colocalization of AAGAA-ClC-6 and ClC-7. DRM analysis showed that ClC-7 floated upwards in a sucrose gradient, but contrary to ClC-6 it did not reach the caveolin-1-positive fractions at the top of the sucrose gradient (data not shown). Finally, N-glycosylation was not important for DRM association of hClC-6, since the triple mutant hClC-6 (AAA-hClC-6: N410A/N422A/N432A) still floated upwards in the sucrose gradient (data not shown).

## Discussion

We have developed a polyclonal antibody against human ClC-6 which has enabled us to study the N-glycosylation profile and the subcellular distribution of hClC-6 both endogenously in the neuronal SH-SY5Y cell line and in transiently transfected COS-1 or Hela cells. Our data are consistent with hClC-6 being a multiply N-glycosylated membrane protein that is targeted to late endosomes in neuronal cells. In transfected COS-1 cells hClC-6 resides in a lipid raft microenvironment and the association with detergent resistant membrane fractions is critically dependent on the positively charged juxtamembranous KKGRR sequence (amino acids 71–75).

The glycosidase profile of hClC-6 (PNGaseF-sensitive, but EndoH resistant) indicates that hClC-6 acquires fully processed, mature N-glycans and therefore that hClC-6 traverses the Golgi apparatus during its biosynthesis. This most likely explains the occasional overlap of ClC-6 with Golgi markers such as Golgin-97 (our observation) or GM-130 [35]. Three asparagine residues (N410, N422 and N432) were identified as N-glycosylation sites, but only two of the three sites are effectively glycosylated in the wild type protein. Whether this is due to steric hindrance, e.g. that the short loop between N410 and N432 can sterically only accommodate two glycan moieties, or to other limiting factors such as the sequence context or associated proteins, is currently not known. An aspartate at the X position in the glycosylation sequon N-X-S/T and a serine at the +2 position reduce the efficiency of N-glycosylation [36]. Thus, the specific sequence context of the hClC-6 glycosylation sites (N410-**D**-**S**; N422-S-**S**; N432-**D**-T) could contribute to their less efficient usage. The amino acid sequence of the predicted exoplasmic region containing N410, N422 and N432 is poorly conserved between the mammalian CLC's. Furthermore, except for ClC-7, all mammalian CLC's contain one or more consensus sites for N-glycosylation in this region (Fig. 3B). N-glycosylation has been experimentally confirmed for human ClC-1 (N430) [37], rat ClC-K1/K2 (N364 and/or N373) [38] and *Xenopus laevis* ClC-5 (N470) [39]. Furthermore, it is very likely that one or more of the predicted sites in human and mouse ClC-3 and human ClC-5 are effectively occupied by a N-glycan, because of the PNGaseF sensitivity of these proteins [40]–[43]. Thus, based on the glycosylation pattern in the K-M region non-glycosylated (e.g. ClC-7), monoglycosylated (e.g. ClC-1) and multiple glycosylated (e.g. ClC-6) isoforms can be distinguished among mammalian CLC's. Because of these sequence and glycosylation variations the exoplasmic K-M region emerges as a highly divergent stretch in mammalian CLC's and is therefore a prime candidate for isoform-specific interactions with extracellular or luminal proteins affecting their function, biosynthesis, degradation and/or sorting. However, there are few experimental data available with respect to the functional significance of glycans in CLC proteins. For the plasma membrane ClC-1, it has been reported that glycosylation-deficient channels are still functional with no effect on the electrophysiological characteristics [38]. N-glycosylation enhances plasma membrane expression of *Xenopus* ClC-5, but it is not required for its endosomal localization [39]. Similarly, non-glycosylated ClC-6 was still able to reach its endosomal location in COS-1 and HeLa cells, but we cannot exclude a role of N-glycosylation in late endosomal sorting. Moreover, the lack of a functional read out system for ClC-6 precludes drawing definitive conclusions about the functional importance of N-glycosylation in ClC-6.

At first sight there seems to be a contradiction between the late endosomal location of endogenous ClC-6 in SH-SY5Y cells and the colocalization with early/recycling endosomal markers when ClC-6 is overexpressed in COS-1, HeLa and SH-SY5Y cells. However, one can draw an interesting comparison with LIMP II, a *bona fide* late endosomal/lysosomal membrane protein [44]. When overexpressed in COS cells, LIMP II induces the appearance of large vesicular structures which are positive for EEA-1 and transferrin receptor [45]. Furthermore, LIMP II overexpression impairs membrane traffic out of the early endosomal compartment. Kuronita *et al.* [45] therefore concluded that LIMP II is sorted to late endosomes/lysosomes via the early endosomal compartment and contributes to the biogenesis of late endosomes/lysosomes by controlling a crucial step in vesicular transport between early and late endosomes. The parallel behaviour of ClC-6 (appearance of enlarged vesicles upon overexpression; endogenous ClC-6 in late endosomes versus overexpressed ClC-6 in early endosomes) prompts several hypotheses with respect to the cell biology of ClC-6. First, it is consistent with the routing of ClC-6 to late endosomes via an early endosomal compartment. In transfected cells exit of ClC-6 from this early compartment seems to be the rate-limiting step, whereas endogenous ClC-6 leaves the early endosomal compartment and is sorted to late endosomes. Second, the enlarged vesicles upon overexpression in COS-1 cells may point to a role of ClC-6 in vesicular transport out of the early compartment. If so, ClC-6 could contribute to late endosomal/lysosomal biogenesis which would explain the lysosomal storage disease phenotype in ClC-6 knock-out mice [18].

It is tempting to interpret the sorting and function of ClC-6 in the context of the Tubular Endosomal Network (TEN) model which has recently been proposed by Bonifacino and Rojas [46]. In this model, the endosomal compartment is divided in a vacuolar part and a tubular extension, the TEN. The vacuolar compartment corresponds to the early endosomal compartment and is the entry site for cargo-containing vesicles derived from the plasma membrane or the trans-Golgi-network (TGN). Proteins that are not destined for lysosomal degradation are separated from the degradative cargo and transported from the early endosomal compartment to the TEN where they are further sorted to specific microdomains from which cargo-loaded vesicles bud off. The microdomains in TEN form exit sites for recycling endosomes that return to the plasmamembrane (e.g. TfR), for retrograde transport vesicles going back to the TGN (e.g. Mannose-6-Phosphate Receptor) and for the lysosomal bypass route via which vesicles containing late endosomal/lysosomal membrane proteins such as LAMP's and LIMP's are sorted to late endosomes [47]. Based on this model, we propose that ClC-6, once it has reached the early endosome, is sorted to the TEN which it leaves via the lysosomal exit site to finally arrive in late endosomes. In transfected cells (COS-1, HeLa, SH-SY5Y) ClC-6 seems to be correctly sorted to TEN as can be deduced from its overlap with early and recycling endosomal markers, but it apparently cannot enter the lysosomal bypass route and therefore does not end up in late endosomes. How would overexpression interfere with the proper sorting of ClC-6? One possibility is that late endosomal delivery of ClC-6 requires an additional factor (a β-subunit as for ClC-Ka/Kb, an adaptor/coat protein required for vesicular transport, …) that is expressed in limiting quantities which are sufficient for correct sorting of the endogenous ClC-6, but insufficient for abundantly overexpressed ClC-6. Alternatively, ClC-6 could be mechanistically involved in the late endosomal sorting (see below) so that overexpression of ClC-6 would block this sorting step.

Given that ClC-6 resides in the lysosomal bypass route, what function would it fulfill? Initially, intracellular CLC's were thought off as Cl^−^ channels facilitating acidification of the organellar lumen by providing an electrogenic shunt for the lumen-positive membrane potential generated by the V-type H^+^-pump [12]. However, loss of ClC-6 or ClC-7 does not affect the lysosomal pH in respectively ClC6 −/− and ClC-7 −/− mice [48] indicating that ClC-6 and ClC-7 are not essential for lysosomal acidification. Furthermore, intracellular CLC's most likely function as Cl^−^/H^+^-antiporters [5], [6] and they can therefore acidify (and increase the luminal Cl^−^ concentration) or alkalinize (and decrease the luminal Cl^−^ concentration) endosomes depending on the electrical, pH and Cl^−^ gradient across the endosomal membrane. This raises the possibility that intracellular CLC's exert an effect on endosomal traffic and/or endosome/lysosome biogenesis by changing the endosomal pH and/or the luminal Cl^−^ concentration. An alternative, not necessarily mutually exclusive, mechanism is that endosomal CLC's function as pH or Cl^−^ sensors that couple changes in lumenal pH or Cl^−^ to conformational changes in their cytosolic domains which could trigger the recruitment of cytosolic factors to the endosome membrane to control specific steps in vesicular transport. Interestingly, such a pH-sensing function has recently been shown for the V-type H^+^-pump in early endosomes [49]. Also, it has very recent been shown that gating of ClC-0 (i.e. the binding of Cl^−^ in the channel pore) causes a conformational change of its carboxyterminus [50].

Finally, our data show that in transiently transfected COS-1 cells hClC-6 associates with detergent-resistant membrane domains suggesting that ClC-6 segregates to lipid rafts. DRM association may be a common theme for CLC proteins since ClC-2 also concentrates in cholesterol-enriched lipid domains which affects the gating properties of the channel [51]. Moreover, the DRM association critically depends on a positively charged amino acid sequence KKGRR which according to the CLC topology model is located immediately N-terminal of helix B, the first transmembrane segment. The positive charge and the cytosolic, membrane-proximal location of this sequence are reminiscent of the RHRRR sequence that functions as a raft localization marker for the CD4 receptor. Surprisingly, mutating the KKGRR sequence also affected the colocalization of ClC-6 with ClC-7. In cotransfection experiments, wild-type ClC-6 and ClC-7 can be spatially resolved on CSLM, whereas AAGAA-ClC-6 and ClC-7 colocalize to a large extent. Whether the differential sorting of wild type ClC-6 and ClC-7 is a lipid-based mechanism (ClC-6 and ClC-7 seem to associate with different lipid domains), or, alternatively, whether protein interactions involving the KKGRR sequence are the driving component, is not clear. Irrespective of the mechanism our data identify the KKGRR sequence as an important cis-acting element for the correct sorting and delivery of ClC-6. However, additional experiments are needed to verify the DRM association of endogenous ClC-6 and the specific effects exerted by the KKGRR sequence on the endogenous sorting process.

To conclude, we have shown that human ClC-6 is an N-glycosylated protein and the N-glycosylation sites have been identified. We have also found a positively charged motif in the N-terminus of the protein that affects both DRM association and segregation from ClC-7 in transfected COS-1 and HeLa cells. Furthermore, our data suggest that upon overexpression in COS-1 and HeLa cells, ClC-6 does not reach the late endosomal compartment, but is retained in an early endosomal compartment that may correspond to the tubular endosomal network. We propose that endogenous ClC-6 leaves the tubular endosomal network via the lysosomal exit site to finally reach the late endosomes. This model puts ClC-6 at the heart of the late endosomal/lysosomal biogenesis route which could explain the lysosomal storage disease phenotype in ClC-6 knock-out mice [18].

## References

[1] Jentsch TJ, Stein V, Weinreich F, Zdebik AA (2002). Molecular structure and physiological function of chloride channels.. Physiological Reviews.

[2] Estevez R, Boettger T, Stein V, Birkenhager R, Otto E (2001). Barttin is a Cl- channel beta-subunit crucial for renal Cl- reabsorption and inner ear K+ secretion.. Nature.

[3] Lange PF, Wartosch L, Jentsch TJ, Fuhrmann JC (2006). ClC-7 requires Ostm1 as a beta-subunit to support bone resorption and lysosomal function.. Nature.

[4] Accardi A, Miller C (2004). Secondary active transport mediated by a prokaryotic homologue of ClC Cl- channels.. Nature.

[5] Picollo A, Pusch M (2005). Chloride/proton antiporter activity of mammalian CLC proteins ClC-4 and ClC-5.. Nature.

[6] Scheel O, Zdebik AA, Lourdel S, Jentsch TJ (2005). Voltage-dependent electrogenic chloride/proton exchange by endosomal CLC proteins.. Nature.

[7] Accardi A, Walden M, Nguitragool W, Jayaram H, Williams C (2005). Separate ion pathways in a Cl-/H+ exchanger.. J Gen Physiol.

[8] Kornak U, Kasper D, Bosl MR, Kaiser E, Schweizer M (2001). Loss of the ClC-7 chloride channel leads to osteopetrosis in mice and man.. Cell.

[9] Gunther W, Luchow A, Cluzeaud F, Vandewalle A, Jentsch TJ (1998). ClC-5, the chloride channel mutated in Dent's disease, colocalizes with the proton pump in endocytotically active kidney cells.. Proc Natl Acad Sci U S A.

[10] Sakamoto H, Sado Y, Naito I, Kwon TH, Inoue S (1999). Cellular and subcellular immunolocalization of ClC-5 channel in mouse kidney: colocalization with H+-ATPase.. Am J Physiol.

[11] Stobrawa SM, Breiderhoff T, Takamori S, Engel D, Schweizer M (2001). Disruption of ClC-3, a chloride channel expressed on synaptic vesicles, leads to a loss of the hippocampus.. Neuron.

[12] Jentsch TJ, Poet M, Fuhrmann JC, Zdebik AA (2005). Physiological functions of CLC Cl- channels gleaned from human genetic disease and mouse models.. Annu Rev Physiol.

[13] Christensen EI, Devuyst O, Dom G, Nielsen R, Van der Smissen P (2003). Loss of chloride channel ClC-5 impairs endocytosis by defective trafficking of megalin and cubilin in kidney proximal tubules.. Proc Natl Acad Sci U S A.

[14] Brandt S, Jentsch TJ (1995). ClC-6 and ClC-7 are two novel broadly expressed members of the CLC chloride channel family.. FEBS Lett.

[15] Buyse G, Trouet D, Voets T, Missiaen L, Droogmans G (1998). Evidence for the intracellular location of chloride channel (ClC)-type proteins: co-localization of ClC-6a and ClC-6c with the sarco/endoplasmic-reticulum Ca2+ pump SERCA2b.. Biochem J.

[16] Buyse G, Voets T, Tytgat J, De Greef C, Droogmans G (1997). Expression of human pICln and ClC-6 in Xenopus oocytes induces an identical endogenous chloride conductance.. J Biol Chem.

[17] Voets T, Buyse G, Tytgat J, Droogmans G, Eggermont J (1996). The chloride current induced by expression of the protein pICln in Xenopus oocytes differs from the endogenous volume-sensitive chloride current.. J Physiol.

[18] Poet M, Kornak U, Schweizer M, Zdebik AA, Scheel O (2006). Lysosomal storage disease upon disruption of the neuronal chloride transport protein ClC-6.. Proc Natl Acad Sci U S A.

[19] Trouet D, Nilius B, Voets T, Droogmans G, Eggermont J (1997). Use of a bicistronic GFP-expression vector to characterise ion channels after transfection in mammalian cells.. Pflugers Arch.

[20] Ho SN, Hunt HD, Horton RM, Pullen JK, Pease LR (1989). Site-directed mutagenesis by overlap extension using the polymerase chain reaction.. Gene.

[21] Sonnichsen B, De Renzis S, Nielsen E, Rietdorf J, Zerial M (2000). Distinct membrane domains on endosomes in the recycling pathway visualized by multicolor imaging of Rab4, Rab5, and Rab11.. J Cell Biol.

[22] Verboomen H, Wuytack F, De Smedt H, Himpens B, Casteels R (1992). Functional difference between SERCA2a and SERCA2b Ca2+ pumps and their modulation by phospholamban.. Biochem J.

[23] Trouet D, Carton I, Hermans D, Droogmans G, Nilius B (2001). Inhibition of VRAC by c-Src tyrosine kinase targeted to caveolae is mediated by the Src homology domains.. Am J Physiol Cell Physiol.

[24] Dowland LK, Luyckx VA, Enck AH, Leclercq B, Yu AS (2000). Molecular cloning and characterization of an intracellular chloride channel in the proximal tubule cell line, LLC-PK1.. J Biol Chem.

[25] Maxfield FR, McGraw TE (2004). Endocytic recycling.. Nat Rev Mol Cell Biol.

[26] Gorvel JP, Chavrier P, Zerial M, Gruenberg J (1991). Rab5 controls early endosome fusion in vitro.. Cell.

[27] Bucci C, Parton RG, Mather IH, Stunnenberg H, Simons K (1992). The small GTPase rab5 functions as a regulatory factor in the early endocytic pathway.. Cell.

[28] Cantalupo G, Alifano P, Roberti V, Bruni CB, Bucci C (2001). Rab-interacting lysosomal protein (RILP): the Rab7 effector required for transport to lysosomes.. Embo J.

[29] van der Sluijs P, Hull M, Webster P, Male P, Goud B (1992). The small GTP-binding protein rab4 controls an early sorting event on the endocytic pathway.. Cell.

[30] de Wit H, Lichtenstein Y, Kelly RB, Geuze HJ, Klumperman J (2001). Rab4 regulates formation of synaptic-like microvesicles from early endosomes in PC12 cells.. Mol Biol Cell.

[31] Ullrich O, Reinsch S, Urbe S, Zerial M, Parton RG (1996). Rab11 regulates recycling through the pericentriolar recycling endosome.. J Cell Biol.

[32] Ren M, Xu G, Zeng J, De Lemos-Chiarandini C, Adesnik M (1998). Hydrolysis of GTP on rab11 is required for the direct delivery of transferrin from the pericentriolar recycling compartment to the cell surface but not from sorting endosomes.. Proc Natl Acad Sci U S A.

[33] Harder T, Scheiffele P, Verkade P, Simons K (1998). Lipid domain structure of the plasma membrane revealed by patching of membrane components.. J Cell Biol.

[34] Popik W, Alce TM (2004). CD4 receptor localized to non-raft membrane microdomains supports HIV-1 entry. Identification of a novel raft localization marker in CD4.. J Biol Chem.

[35] Suzuki T, Rai T, Hayama A, Sohara E, Suda S (2006). Intracellular localization of ClC chloride channels and their ability to form hetero-oligomers.. J Cell Physiol.

[36] Jones J, Krag SS, Betenbaugh MJ (2005). Controlling N-linked glycan site occupancy.. Biochim Biophys Acta.

[37] Schmidt-Rose T, Jentsch TJ (1997). Transmembrane topology of a CLC chloride channel.. Proc Natl Acad Sci U S A.

[38] Kieferle S, Fong P, Bens M, Vandewalle A, Jentsch TJ (1994). Two highly homologous members of the ClC chloride channel family in both rat and human kidney.. Proc Natl Acad Sci U S A.

[39] Schmieder S, Bogliolo S, Ehrenfeld J (2007). N-glycosylation of the Xenopus laevis ClC-5 protein plays a role in cell surface expression, affecting transport activity at the plasma membrane.. J Cell Physiol.

[40] Schmieder S, Lindenthal S, Ehrenfeld J (2001). Tissue-specific N-glycosylation of the ClC-3 chloride channel.. Biochem Biophys Res Commun.

[41] Weylandt KH, Valverde MA, Nobles M, Raguz S, Amey JS (2001). Human ClC-3 is not the swelling-activated chloride channel involved in cell volume regulation.. J Biol Chem.

[42] Huang P, Liu J, Di A, Robinson NC, Musch MW (2001). Regulation of human CLC-3 channels by multifunctional Ca2+/calmodulin-dependent protein kinase.. J Biol Chem.

[43] Devuyst O, Christie PT, Courtoy PJ, Beauwens R, Thakker RV (1999). Intra-renal and subcellular distribution of the human chloride channel, CLC-5, reveals a pathophysiological basis for Dent's disease.. Hum Mol Genet.

[44] Barriocanal JG, Bonifacino JS, Yuan L, Sandoval IV (1986). Biosynthesis, glycosylation, movement through the Golgi system, and transport to lysosomes by an N-linked carbohydrate-independent mechanism of three lysosomal integral membrane proteins.. J Biol Chem.

[45] Kuronita T, Eskelinen EL, Fujita H, Saftig P, Himeno M (2002). A role for the lysosomal membrane protein LGP85 in the biogenesis and maintenance of endosomal and lysosomal morphology.. J Cell Sci.

[46] Bonifacino JS, Rojas R (2006). Retrograde transport from endosomes to the trans-Golgi network.. Nat Rev Mol Cell Biol.

[47] Peden AA, Oorschot V, Hesser BA, Austin CD, Scheller RH (2004). Localization of the AP-3 adaptor complex defines a novel endosomal exit site for lysosomal membrane proteins.. J Cell Biol.

[48] Kasper D, Planells-Cases R, Fuhrmann JC, Scheel O, Zeitz O (2005). Loss of the chloride channel ClC-7 leads to lysosomal storage disease and neurodegeneration.. Embo J.

[49] Hurtado-Lorenzo A, Skinner M, El Annan J, Futai M, Sun-Wada GH (2006). V-ATPase interacts with ARNO and Arf6 in early endosomes and regulates the protein degradative pathway.. Nat Cell Biol.

[50] Bykova EA, Zhang XD, Chen TY, Zheng J (2006). Large movement in the C terminus of CLC-0 chloride channel during slow gating.. Nat Struct Mol Biol.

[51] Hinzpeter A, Fritsch J, Borot F, Trudel S, Vieu DL (2007). Membrane cholesterol content modulates ClC-2 gating and sensitivity to oxidative stress.. J Biol Chem..

[52] Ignoul S, Eggermont J (2005). CBS domains: structure, function, and pathology in human proteins.. Am J Physiol Cell Physiol.

[53] Dwek RA, Edge CJ, Harvey DJ, Wormald MR, Parekh RB (1993). Analysis of glycoprotein-associated oligosaccharides.. Annu Rev Biochem.

